# Supplementary material for: Asymmetric Synthesis of Quaternary Hydantoins via a Palladium-Catalyzed Aza-Heck Cyclization
Source: J Am Chem Soc. 2025 Nov 14;147(49):44692–8. doi: 10.1021/jacs.5c16022 (PMC12703750; doi:10.1021/jacs.5c16022)
Supplement: Supplementary file 2 [file ja5c16022_si_002.zip › All NMR FID Files/15/15_AllNMR/2/pdata/1/email_EM04-185-dry_2_1.pdf]

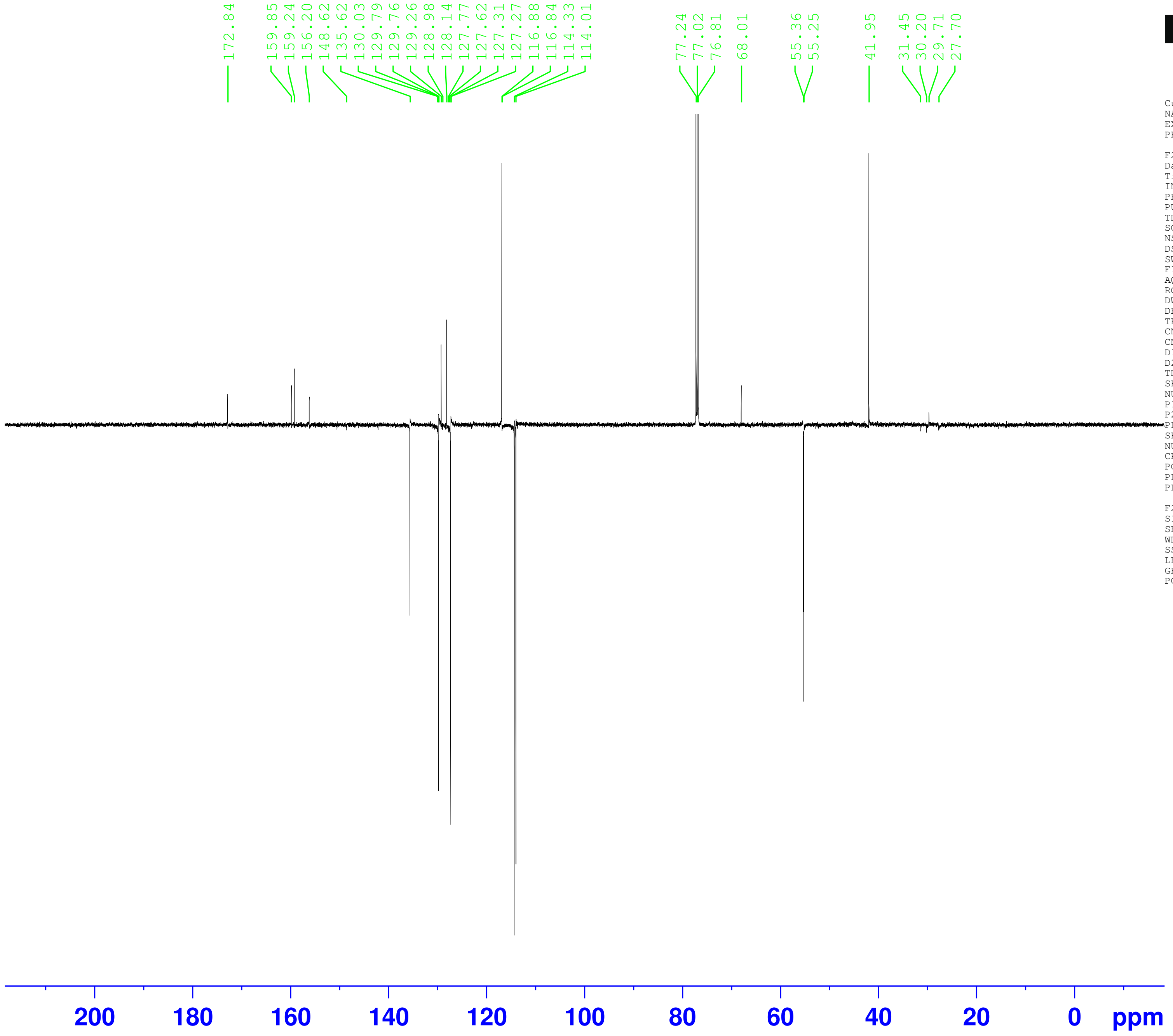


```
Current Data Parameters
NAME          EM04-185-dry
EXPNO                    2
PROCNO                   1

F2 - Acquisition Parameters
Date_             20231006
Time                  8.29 h
INSTRUM   Avance Neo 600 at University of Delaware
PROBHD    Z185170_0001 (
PULPROG               jmod
TD                   65536
SOLVENT              CDCl3
NS                    1598
DS                       4
SWH              35714.286 Hz
FIDRES            1.089913 Hz
AQ               0.9175040 sec
RG                     101
DW                  14.000 usec
DE                   19.25 usec
TE                   298.0 K
CNST2          145.0000000
CNST11           1.0000000
D1              2.00000000 sec
D20             0.00689655 sec
TD0                      1
SFO1           150.9178988 MHz
NUC1                   13C
P1                   11.78 usec
P2                   23.56 usec
PLW1          106.00000000 W
SFO2           600.1324005 MHz
NUC2                    1H
CPDPRG[2           waltz65
PCPD2                70.00 usec
PLW2            6.90000010 W
PLW12           0.09034800 W

F2 - Processing parameters
SI                   32768
SF             150.9028085 MHz
WDW                     EM
SSB                      0
LB                    1.00 Hz
GB                       0
PC                    1.40
```
